# Preliminary Evaluation of Supercritical Carbon Dioxide Extracted Dabai Pulp Oleoresin as a New Alternative Fat

**DOI:** 10.3390/molecules26185545

**Published:** 2021-09-13

**Authors:** Noor Atiqah Aizan Abdul Kadir, Azrina Azlan, Faridah Abas, Intan Safinar Ismail

**Affiliations:** 1 Department of Nutrition, Faculty of Medicine and Health Sciences, Universiti Putra Malaysia (UPM), Serdang 43400, Selangor, Malaysia; atiqahaizan@yahoo.com; 2 Research Centre for Excellence for Nutrition and Non-Communicable Disease, Faculty of Medicine and Health Sciences, Universiti Putra Malaysia (UPM), Serdang 43400, Selangor, Malaysia; 3 Halal Products Research Institute, Universiti Putra Malaysia (UPM), Serdang 43400, Selangor, Malaysia; 4 Department of Food Science, Faculty of Food Science and Technology, Universiti Putra Malaysia (UPM), Serdang 43400, Selangor, Malaysia; faridah_abas@upm.edu.my; 5 Department of Chemistry, Faculty of Science, Universiti Putra Malaysia (UPM), Serdang 43400, Selangor, Malaysia; safinar@upm.edu.my

**Keywords:** alternative fat, oleoresin, quality parameters, supercritical carbon dioxide extraction

## Abstract

There has been growing interest among food scientists in producing a toxin-free fat as an end product with varying physical or nutritional properties of interest to the food industry. Oleoresin is a rich source of bioactive compounds which consumers can easily add to a large variety of food. Dabai (*Canarium odontophyllum*) pulp oleoresin (DPL) was extracted using supercritical carbon dioxide (SC-CO_2_) extraction, a green extraction technology. This study investigates the quality of SC-CO_2_ extracted DPL in discovering its potential as a new alternative fat. The extraction experiment was carried out at a pressure of 40 MPa and a temperature of 40 °C. DPL is a saturated fatty acid (SFA)-rich fat due to its high SFA composition (47.72 ± 0.01%). In addition, the low content of peroxide value (PV) (5.60 ± 0.09 mEq/kg) and free fatty acids (FFA) (3.40 ± 0.03%) indicate the quality and stability of DPL for various applications besides food consumption. DPL also has a low slip melting point (SMP) (20.20 ± 0.03 °C), and HPLC-FID revealed that DPL contained 0.13 ± 0.02 mg/100 g of vitamin E (α-tocopherol), indicating its potential application as a solid fat with a bioactive compound. This present work demonstrates the possible prospect of DPL in the formulation of end products for food industries.

## 1. Introduction

*Canarium odontophyllum*, locally known as dabai, is a speciality and indigenous seasonal fruit of Sarawak, Malaysia. Interestingly, dabai fruit is uniquely known as ‘Sarawak olive’ due to its resemblance in physical appearance, texture, and flavour with olive. The Sarawak local community eats the fruits with sugar or soy sauce after being soaked in warm water for 15 min. The fruits are harvested and sold only in the local market [1]. Dabai is a nutritious and tasty fruit with an avocado-like taste. The fresh fruit has yellowish oily pulp and dark purple peel. The edible portion of the dabai fruit, dabai pulp, has a high phenolic content level, 267.0 ± 4.24 mg GAE/100 g of total phenolic content [2]. In addition, the fruit contains protein, fat, and carbohydrate, making it an excellent food [3]. Furthermore, dabai pulp has the highest fat, protein, and crude fibre contents compared with other indigenous fruits [4].

Our previous studies have available data on dabai pulp’s potential as a functional food [5,6,7,8]. Dabai pulp oil (DPO) is a saturated fatty acid (SFA)-rich oil. DPO showed a hypocholesterolemic effect by reducing total cholesterol (TC) and triglyceride (TG) in high cholesterol diet-fed rats [6]. Meanwhile, defatted dabai pulp (DDP) showed beneficial effects in hypercholesterolemic rats by reducing TC, TG, and low-density lipoprotein (LDL) levels. DDP also has a good effect against oxidative stress by increasing the antioxidant enzymes and lowering inflammatory markers in hypercholesterolemic rats. Interestingly, DDP showed a therapeutic ability via choline metabolism in the metabolism dysfunction caused by hypercholesterolemia [8].

The concern over the quality and safety of food products and regulations of the residual level of solvents has driven the food industry to develop a new alternative to traditional solvents for extraction, fractionation, and isolation of active ingredients [9]. As a result, supercritical fluid extraction (SFE) is state-of-the-art in extracting and fractionating desired bioactive ingredients. SFE is essential in the food, pharmaceutical, and cosmetic industries because the extraction produces a high purity toxin-free product without sample degradation [10]. Furthermore, this novel technology made the extraction of desired essential products such as oleoresin very simple, which consumers can easily add to a large variety of food [11].

Oleoresins are composed of two fractions; the volatile oil and the non-volatile components. The latter is mainly resins, waxes, lipophilic components, and colour. Oleoresin is a rich source of bioactive compounds with various applications in the food industry [12]. Previously, SFE extracted oleoresin from various plant sources such as fruits, spices, and herbs. For example, the SFE of tomato oleoresin (extracted at a pressure of 45 MPa, and temperature of 80 °C) showed an intense red colour [13]. The tomato oleoresin finds its application in the food industry to substitute for tomato fruit, colouring, and flavouring agent [14]. Meanwhile, marigold flower oleoresin (SFE extraction under the pressure of 14–34 MPa, and temperature of 40 °C) is a yellowish-orange colour known as a natural colouring and flavouring agent in the food industry [15]. The SFE extracted rosemary oleoresin (under the pressure of 20–40 MPa, and at temperatures of 40–60 °C) [16] is a natural preservative in food, such as pork sausage and sunflower oil [12].

Previously, we successfully extracted DPL by using SC-CO_2_ under the pressure of 40 MPa and a temperature of 40 °C [17]. Further, we also demonstrated the potential use of DPL as a cocoa butter substitute in making cocoa bars. The inclusion of 20% DPL exhibited good nutritional composition (high in protein and total available carbohydrates) [17]. Today, the food application of SC-CO_2_ extracted DPL is relatively new, and there are no published data available regarding the quality parameters of DPL. This work aims to evaluate some quality parameters of SC-CO_2_ extracted DPL in promoting the potential of DPL as a new alternative fat.

## 2. Results and Discussion

### Quality Parameters of DPL

Currently, SC-CO_2_ is the only extraction technique used to extract DPL in Malaysia. This work presents the preliminary quality evaluation of SC-CO_2_ extracted DPL to emphasise its potential as an alternative fat. This study is the first to evaluate the fatty acid composition (FAC), PV, FFA, SMP, and vitamin E (α-tocopherols) in SC-CO_2_ extracted DPL, which is the by-product of the SC-CO_2_ extracted DPO. Previously, we demonstrated the quality of DPO as a new alternative fat and its efficacy in hypercholesterolemic rats [6].

DPL comprises 47.72 ± 0.01% of SFA, 40.56 ± 1.05% of monounsaturated fatty acid (MUFA) and 13.48 ± 1.28% of polyunsaturated fatty acid (PUFA). The main component of DPL was palmitic acid (41.62 ± 0.01%), followed by oleic acid (39.68 ± 1.21) and linoleic (cis) acid (12.85 ± 1.25) (Table 1). Due to the high composition of palmitic acid, DPL is an SFA-rich fat and can further be grouped into a palmitic acid subclass. Interestingly, all these main components in DPL were comparable with crude palm oil (CPO). CPO contained a balanced ratio of unsaturated and saturated fatty acid; 37.4–44.1% oleic acid (MUFA), 8.7–12.5% linoleic acid (PUFA), 39.2–45.8% palmitic acid and 3.7–5.1% stearic acid (SFA) [18]. The similarity is characterised by a near equal percentage of unsaturated and saturated fatty acid in DPL; 39.68% oleic acid (MUFA), 12.85% linoleic acid (PUFA), 41.62% palmitic acid, and 4.31% stearic acid (SFA). The investigation of DPL indirectly adds variety to the types of alternative fat for commercialisation. SC-CO_2_ extracted DPL may be easily fractionated into a solid fat and a liquid oil because of its near-equal saturated to unsaturated fatty acid ratio. These fractions can then be applied in various quantities to serve as raw materials for margarine manufacturing. Nowadays, several kinds of margarine, for example, hard margarine, soft table margarine and puff pastry margarine, are needed to suit the demands of the food industries [19].

There was a decrease in the percentage of shorter-chain fatty acid components (C8–C14) with a longer extraction time. In contrast, the percentage of long-chain fatty acid components (C16–C18:2) increased with time [20]. In this study, the DPL was extracted for 2 h, which provides favourable conditions for the fatty acid in DPL with a chain longer than C16 to be extracted. Previously, it was shown that lower pressure (20.7–27.6 MPa) resulted in a decreased extraction of shorter-chain fatty acid components. Meanwhile, higher long-chain fatty acid components were recovered at higher pressures (34.5–48.3 MPa) [21]. A higher pressure of SC-CO_2_ was used in this study (40 MPa), explaining a higher percentage of palmitic acid (C16), oleic acid (C18:1n9c) and linoleic (cis) acid (c18:2n6c) in DPL. The temperature of the SC-CO_2_ extraction method (40–60 °C) had a favourable effect on the number of total lipids recovered, most likely because of the increased lipids’ vapour pressure. Moreover, a higher percentage of long-chain fatty acid components were recovered with increased temperature. In fact, at greater pressures, this effect was more noticeable [22].

Regarding the extraction time, pressure, and temperature, the same trend was observed in the previous literature for the influence of these parameters on the fatty acid composition. Fattori et al. [23] used SC-CO_2_ to fractionate the lipids in canola seed extracts and found that the later fractions were richer in C22 and C24 fatty acids than the early fractions. Fatouh et al. [24] demonstrated that SC-CO_2_ extraction effectively created buffalo butter oil fractions with vastly different characteristics. Fractionation was carried out at temperatures of 50 and 70 °C and pressures ranging from 109 to 401 bar. From the first to the last fractions, short-chain fatty acids (C4–C8), medium-chain fatty acids (C10–C14), and saturated fatty acids decreased, while long-chain fatty acids (C16–C18:3) and unsaturated fatty acids increased.

Ragunath et al. [25] investigated the fractionation of fatty oil constituents of C6, C12, C16, and C18:1 using SC-CO_2_ at 40–80 °C and 300 bar. The scientists reported that the components’ solubility increased with pressure; however, the solubility declined as the carbon number grew under constant pressure. Meanwhile, Hassan et al. [20] reported that palm kernel oil (P.K.O.) solubility in SC-CO_2_ decreased with the temperature at lower pressures of 20.7 and 27.6 MPa, but increased at higher pressures of 34.5, 41.4, and 48.3 MPa. Additionally, the authors discovered that the early fractions were high in short-chain fatty acids, whereas the latter fractions were high in long-chain fatty acids and unsaturated fatty acids. Additionally, the P.K.O. short-chain fatty acids are readily soluble in SC-CO_2_.

An edible fats’ quality is affected by quality parameters such as PV and FFA. PV is an indicator of peroxide’s formation at the early oxidation stage. Determining PV is a critical approach to ensure the quality of the extracted fat [26]. As presented in Table 2, the PV of DPL is 5.60 ± 0.09 mEq/kg, which is higher than PV in DPO (4.97 ± 0.00 mEq/kg) [6]. However, the PV in DPL is within the range of other SFA-rich fat such as CPO (0.00–10.40 mEq/kg) [27]. SFE extracted CPO shows a lower PV value than solvent-extracted CPO and commercial CPO [28]. Fat extracted using SFE offers stability benefits due to lower PV; a lesser chance of an oxidative reaction occurring while executing carbon dioxide in a closed system without oxygen [29]. All food scientists should emphasise producing fat with PV as low as possible because a low PV value indicates an excellent fat quality. A general rule is that PV should not be above 10–20 mEq/kg of fat [30]. It is important to note that the PV of DPL is less than 10 mEq/kg of fat, indicating that DPL has good stability towards oxidation and can be considered safe for human consumption.

Further, the FFA of DPL is 3.40 ± 0.03%, which is higher than FFA in DPO (2.57 ± 0.03%) [6], yet within the range of other SFA-rich fat such as CPO (maximum content of FFA in CPO is 5%) [18]. The quality of raw material utilised in fats production and storage settings is an essential indicator of the FFA value in fats [31]. Dabai fruits were placed in the chiller (4 °C) as soon as they arrived and processed the next day to avoid deterioration. Fruits that deteriorated have a high FFA content in their fats [32]. Additionally, DPL was kept in an opaque white plastic container. The container’s opaque quality minimised light absorption by the stored oil, lowering the risk of FFA accumulation and rancidity [32].

Additionally, the lower amount of FFA in DPL can be due to the solubility of polar lipids in SC-CO_2_. The solubility of polar lipids are low in SC-CO_2_; FFA owning a negative charge at the carboxyl end group were not extracted by SFE [29], as it was proven that SC-CO_2_ extracted CPO shows lower FFA than the conventional technique [19]. As the amount of PUFA increases, so does the creation of primary oxidation products [33]. We observed that PUFA in DPL was slightly higher than in DPO [6]; this explained a higher PV and FFA in DPL than DPO. However, given the quality of PV and FFA values, the sampling of dabai fruit and the storage condition protected the fat from deterioration.

The SMP of DPL in this study was 20.20 ± 0.03 °C, which is lower than the SMP in palm-based margarine (43 °C) [34], cocoa butter (33.01 ± 0.00 °C), and hydrogenated fat (38 ± 0.5 °C) [35]. The redistribution and alteration of the fatty acid within the triacylglycerol molecules affects the fat’s melting attributes. The melting properties of fat are essential qualities in food product production, especially margarine [36]. At low temperatures, margarine that has low solids can be spreadable directly to food from refrigerators. Additionally, fat blends should be wholly melted below 37 °C for an excellent oral meltdown [37]. Therefore, this study demonstrated DPL’s potential as a table margarine as the SMP is below 37 °C. Previously, we also demonstrated that 20% of DPL in cocoa bars showed no significant difference in colour, aroma, flavour, brittleness, aftertaste, and overall acceptability compared with dark chocolate (40% cocoa butter). Hence, this research suggested that DPL showed potential use as a cocoa butter substitute in cocoa bars [17].

In this study, DPL contained 0.13 ± 0.02 mg/100 g of vitamin E (α-tocopherol). There are no existing data on the vitamin E content in DPL. However, data on vitamin E in dabai pulp fat extract was available for DPO and dabai kernel oil (DKO). Previously, vitamin E was not detected in the petroleum-ether extracted DPO. However, about 12.04 mg/100 g oil of vitamin E was found in DKO, which is higher than in olive oil (1.04 mg/100 g) [1], almond oil (3.1 mg/100 g), and cashew nut oil (5.1 mg/100 g) [38]. In addition, it was shown that vitamin E (α-tocopherols) in different sources such as seed, oil, leaves, and husk extracted using SC-CO_2_ was higher than the value obtained by solvent extraction [39]. A previous study reported a higher concentration of vitamin E (α-tocopherols) in Bilberry seed oil extracted by SC-CO_2_ (18.1 mg/100 g oil) compared with Bilberry seed oil extracted by hexane (4.7 mg/100 g oil) [40].

Vitamin E is fat-soluble and located inside the cell membrane and builds complexes alongside lysophospholipids and free fatty acids [41]. Vitamin E is concentrated inside domains that are rich in lipids, thus preventing the lipids from oxidation. When the fruits are dried and ground, the cell membranes and walls are broken. By applying SC-CO_2_, the oil bodies are quickly extracted [42]. It has been well-documented that vitamin E (α-tocopherol) plays a significant role as an antioxidant in membranes and lipoproteins within the plasma and food system. Due to its antioxidant potential and function at the molecular level, dietary α-tocopherol benefited against cardiovascular disease risk [40]. In this study, SC-CO_2_ at 40 MPa and 40 °C for the DPL extraction was advantageous for recovering vitamin E content in DPL

The present study showed that SC-CO_2_ can be used in extracting SFA-rich DPL. The ease of operating conditions of SFE leads to the prospect of applying fractionation via SC-CO_2_ extraction [9]. The fractionation of DPL based on its FAC plays a significant role in producing end products with varying physical or nutritional properties of interest to the food industry. Based on these study results, DPL is safe for human consumption and shows excellent potential as a new margarine and cocoa butter substitute containing bioactive compounds (vitamin E).

## 3. Materials and Methods

### 3.1. SC-CO_2_ Extraction of DPL

We collected fresh dabai fruits from Sarikei Sarawak, Malaysia. All samples collected were matured fruits. The fruits’ variety and maturity identification was conducted by the Agriculture Research Centre (A.R.C.) research officers from Semongok, Sarawak, Malaysia, and the herbarium voucher specimens (S 64872) of these fruits were deposited. The preparation of dabai pulp was performed based on the method described in our previous study [5].

**Figure 1:**
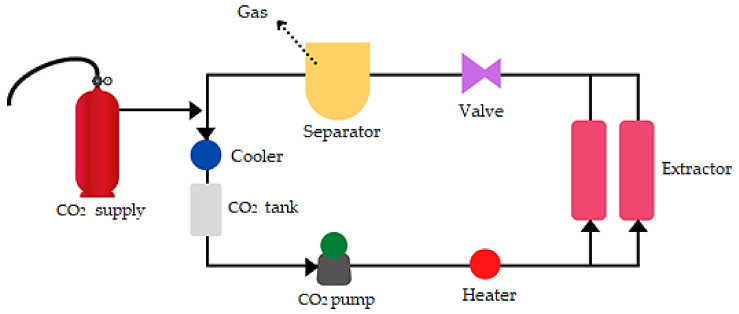
Schematic diagram of the SC-CO_2_ extraction system of DPL.

**Figure 2:**
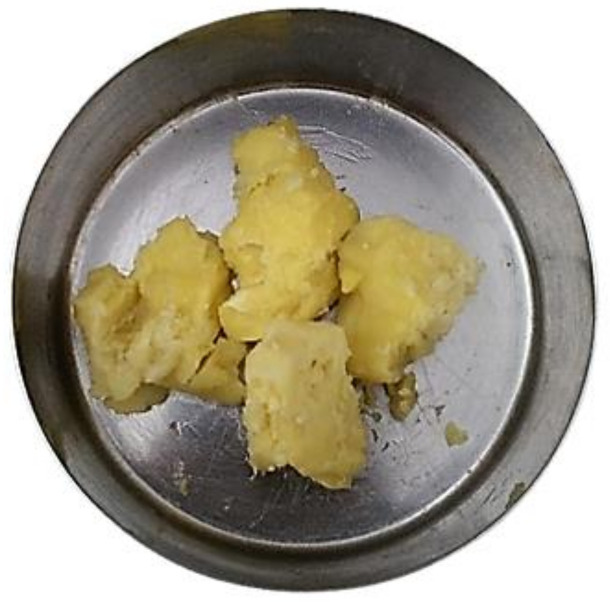
SC-CO_2_ extracted DPL.

Dabai pulp oleoresin (DPL) was extracted by using the SC-CO_2_ extraction technique (Figure 1). The extraction condition was performed at a pressure of 40 MPa and a temperature of 40 °C for 2 h. The extraction protocol was executed as described in our previous study [17]. DPL (Figure 2) was stored in an opaque white plastic container and stored in a chiller at low temperature (4 °C) until further use.

### 3.2. Quality Analysis of DPL

#### 3.2.1. Determination of FAC

The FAC of DPL was analysed by GC after derivatisation of the oils to FAME according to the IUPAC standard method IUPAC 2.301 protocol [43]. An HP 5890 series II plus gas chromatography system (Agilent Technologies, Waldbronn, Germany) equipped with a split/splitless injector, DB 225 capillary column (30 m × 0.25 mm × 0.25 mm) and a flame ionisation detector (FID) were used. Hydrogen was used as a carrier gas, and the flow rate was 1.3 mL/min. The detector temperatures were 240 °C. The oven temperature was from 30 to 250 °C. Overall, the run time was 30 min. Chromatography data were recorded and integrated using the Chemstations software. The fatty acid content in DPL was quantified and determined based on FAMEs (Supelco 37 Component FAME Mix, Cat. No: 18919, Sigma Aldrich, Saint Louis, MO, USA).

#### 3.2.2. Determination of PV and FFA

The determination of PV in DPL was carried out using the AOAC official method 965.33 [44]. Meanwhile, the determination of FFA in DPL was conducted using the MPOB test methods, p2.5:2004 [45]. The PV and FFA protocols were executed as described in our previous study [6].

#### 3.2.3. Determination of SMP

The SMP of DPL was analysed by using the MPOB test method, p4.2:2004 [45]. Three clean capillary tubes were dipped to a depth of 10 mm in a melted DPL sample. After that, the tubes were refrigerated until the DPL sample hardened before being placed in a test tube and held in a beaker of water equilibrated at 10 °C for 16 h in a thermostat water bath (Huber, Offenburg, Germany). After that, the capillary tubes were removed from the test tube and fastened to a thermometer with a rubber band; the lower ends of the tubes were level with the bottom of the thermometer’s mercury bulb. The thermometer was suspended in 400 ml of boiled distilled water in a beaker to a depth of 30 mm. The thermostat water bath was set to a temperature of 8–10 °C below the expected SMP of the DPL sample. A magnetic stirrer was used to agitate the water bath, next the heat was applied at a rate of 1 °C/min before being lowered to 0.5 °C/min. The SMP of DPL is the temperature at which the DPL sample in the tubes began to melt and become transparent. The average temperature of all capillary tubes was recorded as the SMP.

#### 3.2.4. HPLC-FLD Analysis of Vitamin E as α-Tocopherol

The analysis of vitamin E in DPL was analysed according to the AOAC 18th edition, 971.30 α-tocopherol and α-tocopherol acetate in foods and feeds [46]. Vitamin E as α-tocopherol in DPL was analysed using reverse-phase high-performance liquid chromatography (HPLC, Agilent Technologies, Waldbronn, Germany). The 1260 Infinity II fluorescence detector was used, the excitation wavelength was 296 nm, and the emission wavelength was 330 nm. The solvent gradient (pump a: methanol: deionised water; 95:5 *v*/*v*, pump b: deionised water) (Thermo Fisher Scientific, MA, USA) was applied to a C18; 5 μm, reversed-phase column; 4.6 × 150 mm ID (Merck KGaA, Darmstadt, Germany) as follows: 0 min, 95% A; 5 min, 95% A; 5.50 min, 97% A; 16 min, 95% A; 25%, 95% A. The flow rate was 1 mL/min. The volume injected was 10 μL. The quantification of vitamin E as α-tocopherol was carried out by injecting standard working solution and determining vitamin E’s retention time (α-tocopherol). The quantitative assay was achieved using an external calibration curve of the four-point standard working solution of vitamin E (α-tocopherol) (Chromatography grade; Merck KGaA, Darmstadt, Germany).

## 4. Conclusions

SC-CO_2_ extraction is a novel technique that can be applied in producing toxin-free SFA-rich DPL. In the future, oleoresin extraction from the dabai kernel should be investigated as the dabai kernel is rich in vitamin E. Additionally, an assessment of oleoresin standards such as volatile oil content, refractive index, and optical rotation should be conducted in DPL to meet the food industry’s specific product requirements.

## Figures and Tables

**Table 1:** Fatty acid composition in DPL.

Fatty Acids		% ^1^
C8	caprylic	0.05 ± 0.00
C10	capric	0.01 ± 0.00
C11	undecanoic	0.02 ± 0.01
C12	lauric	0.77 ± 0.00
C14	myristic	0.28 ± 0.00
C15	pentadecanoic	0.04 ± 0.01
C16	palmitic	41.62 ± 0.01
C17	heptadecanoic	0.11 ± 0.01
C18	stearic	4.31 ± 0.00
C20	arachidic	0.10 ± 0.00
C21	henicosanoic	0.03 ± 0.00
C22	behenic	0.2 ± 0.00
C23	tricosanoic	0.11 ± 0.00
C24	lignoceric	0.09 ± 0.00
**Saturated fatty acids**		**47.72 ± 0.01**
C15:1	cis-10-pentadecenoic	0.04 ± 0.01
C16:1	palmitoleic	0.63 ± 0.02
C17:1	cis-10-heptadecanoic	0.03 ± 0.00
C18:1n9c	oleic	39.68 ± 1.21
C20:1n9	cis-11-eicosenoic	0.07 ± 0.00
C22:1n9	erucic	0.02 ± 0.01
C24:1	nervonic	0.11 ± 0.13
**Monounsaturated fatty acids**		**40.56 ± 1.05**
c18:2n6c	linoleic (cis)	12.85 ± 1.25
C18:3n6	Υ-linolenic	0.13 ± 0.02
C18:3n3	a-linolenic	0.51 ± 0.01
**Polyunsaturated fatty acids**		**13.48 ± 1.28**

^1^ % in fat indicates the mean of weight (%) in total fatty acids. Values are the mean ± SD (*n* = 3).

**Table 2:** Peroxide value, free fatty acid, slip melting point and vitamin E in DPL.

Quality Analysis	
Peroxide value (mEq/kg)	5.60 ± 0.09
Free fatty acid (%, as in oleic acid)	3.40 ± 0.03
Slip melting point (°C)	20.20 ± 0.03
Vitamin E (α-tocopherol) (mg/100 g)	0.13 ± 0.02

Results are given as mean ± S.D. (*n* = 3).

## Data Availability

The data presented in this study are available on request from the corresponding author.

## Sample Availability

Not available.
